# Comparative genomics of a cannabis pathogen reveals insight into the evolution of pathogenicity in *Xanthomonas*

**DOI:** 10.3389/fpls.2015.00431

**Published:** 2015-06-16

**Authors:** Jonathan M. Jacobs, Céline Pesce, Pierre Lefeuvre, Ralf Koebnik

**Affiliations:** ^1^Institut de Recherche pour le Développement – Cirad – Université Montpellier, Interactions Plantes Microorganismes EnvironnementMontpellier, France; ^2^Department of Applied Microbiology, Earth and Life Institute, Université Catholique de LouvainLouvain-la-Neuve, Belgium; ^3^Pôle de Protection des Plantes, Cirad, UMR Peuplements Végétaux et Bioagresseurs en Milieu TropicalSaint-Pierre, Ile de la Réunion, France

**Keywords:** comparative genomics, *Xanthomonas*, hemp, cell-wall degrading enzymes, type II secretion system, type III secretion system, *hrp genes*, PIP box

## Abstract

Pathogenic bacteria in the genus *Xanthomonas* cause diseases on over 350 plant species, including cannabis (*Cannabis sativa* L.). Because of regulatory limitations, the biology of the *Xanthomonas*-cannabis pathosystem remains largely unexplored. To gain insight into the evolution of *Xanthomonas* strains pathogenic to cannabis, we sequenced the genomes of two geographically distinct *Xanthomonas* strains, NCPPB 3753 and NCPPB 2877, which were previously isolated from symptomatic plant tissue in Japan and Romania. Comparative multilocus sequence analysis of housekeeping genes revealed that they belong to Group 2, which comprises most of the described species of *Xanthomonas*. Interestingly, both strains lack the Hrp Type III secretion system and do not contain any of the known Type III effectors. Yet their genomes notably encode two key Hrp pathogenicity regulators HrpG and HrpX, and *hrpG* and *hrpX* are in the same genetic organization as in the other Group 2 xanthomonads. Promoter prediction of HrpX-regulated genes suggests the induction of an aminopeptidase, a lipase and two polygalacturonases upon plant colonization, similar to other plant-pathogenic xanthomonads. Genome analysis of the distantly related *Xanthomonas maliensis* strain 97M, which was isolated from a rice leaf in Mali, similarly demonstrated the presence of HrpG, HrpX, and a HrpX-regulated polygalacturonase, and the absence of the Hrp Type III secretion system and known Type III effectors. Given the observation that some *Xanthomonas* strains across distinct taxa do not contain *hrpG* and *hrpX*, we speculate a stepwise evolution of pathogenicity, which involves (i) acquisition of key regulatory genes and cell wall-degrading enzymes, followed by (ii) acquisition of the Hrp Type III secretion system, which is ultimately accompanied by (iii) successive acquisition of Type III effectors.

## Introduction

Plant pathogenic bacteria in the genus *Xanthomonas* collectively cause major losses worldwide on over 350 plant species, including crops such as banana, tomato, pepper, sugar cane, and many cereals. Over 20 *Xanthomonas* species are divided into two main phylogenetic groups based on 16S rDNA and *gyrB* sequence analysis (Hauben et al., 1997; Parkinson et al., 2007) and subdivided into pathovars loosely corresponding to host specificity. Group 1, also known as the early branching group, comprises highly diverse *Xanthomonas* species including important sugarcane and cereal pathogens (e.g., *Xanthomonas sacchari*, *Xanthomonas albilineans* and *Xanthomonas translucens*, Hauben et al., 1997; Parkinson et al., 2007). Group 2, the largest and best-described group, includes species such as *Xanthomonas oryzae*, *Xanthomonas citri*, *Xanthomonas vasicola*, *Xanthomonas euvesicatoria*, *Xanthomonas axonopodis* and *Xanthomonas campestris* (Hauben et al., 1997; Parkinson et al., 2007). This diverse genus of bacteria infects and associates with many plant hosts, but individual strains typically possess very restricted host ranges limited to a single genus.

*Xanthomonas* spp. employ a suite of virulence factors to colonize plant tissue, including adhesins, cell wall-degrading enzymes, extracellular polysaccharide and protein secretion systems (Büttner and Bonas, 2010). The Hrp (hypersensitive response and pathogenicity) Type III secretion system (T3SS) is a major virulence trait found in most pathogenic *Xanthomonas* spp. and serves as a molecular syringe to deliver effector proteins into host cells to suppress defenses and modulate plant physiology to promote pathogen growth (White et al., 2009). Plants also evolved resistance proteins that recognize pathogen avirulence effectors and inhibit infection often via a hypersensitivity response (HR), a form of programmed cell death (Bent and Mackey, 2007). A majority of sequenced pathogenic *Xanthomonas* strains have limited host ranges likely due to the plant recognition of Type III (T3)-secreted avirulence effectors (White et al., 2009). In *Xanthomonas* spp., HrpX, an AraC-type regulator, is the transcriptional activator of the genes encoding the T3SS and many of its associated effectors (Koebnik et al., 2006; Tang et al., 2006). HrpG, an OmpR-family and major pathogenicity regulator, positively regulates expression of *hrpX* (Tang et al., 2006). Mutant strains lacking either *hrpX* and *hrpG* are unable to activate expression of the T3SS and thus are non pathogenic (Wengelnik et al., 1996; Tang et al., 2006; Mole et al., 2007). The importance of the T3SS and many T3-secreted effectors during infection is heavily studied, but the evolutionary history of the acquisition of genes encoding the T3SS, associated T3-secreted effectors and regulators, HrpX and HrpG, remains unclear.

Hemp or cannabis (*Cannabis sativa* L.) is a major, global cash crop with many applications such as seed for human consumption, oil, fiber for clothing or ropes, pulp for paper, plastic and composite material (www.hemp.com). Since 2010 worldwide hemp production has increased, and recent surges of hemp production in the United States, China, Australia, Canada, and many other countries have made hemp a multi-million dollar industry (www.hemp.com, www.faostat.fao.org). A draft genome is now available for *C. sativa* cv. Purple Kush (van Bakel et al., 2011), potentially providing a base for molecular and evolutionary understanding of this plant species. Hemp plant production is limited by bacteria, fungi, nematodes, and viruses (McPartland et al., 2000), but because of regulatory constraints, little is known about hemp diseases such as bacterial leaf spot of cannabis caused by *Xanthomonas* species.

Symptoms associated with *Xanthomonas* bacterial leaf spot include water-soaking lesions followed by necrosis accompanied by a yellow halo (Severin, 1978; Netsu et al., 2014). The host range of these *Xanthomonas* strains appears to be quite large unlike most xanthomonads (Severin, 1978; Netsu et al., 2014). Under laboratory conditions, these bacteria caused symptoms on a wide range of plants including cannabis, tomato, mulberry, geranium and *Ficus erecta* (Severin, 1978; Netsu et al., 2014). These strains further trigger an HR on tobacco, but do not elicit any response after inoculation on common bean (Severin, 1978; Netsu et al., 2014). The factors that contribute to pathogenicity and host range of cannabis-infecting *Xanthomonas* are unknown.

To gain insight into the evolution and pathogenicity of bacterial pathogens of cannabis, we sequenced two geographically distinct *Xanthomonas* strains, NCPPB 3753 and 2877, which were previously isolated from symptomatic hemp leaf tissue from Japan and Romania, respectively (Severin, 1978; Netsu et al., 2014). We tested their ability to infect barley, a previously unreported, compatible monocot host. We determined with comparative whole genome analysis based on average nucleotide identity (ANI) and multilocus sequence analysis (MLSA) the relationship of these cannabis strains to each other and other xanthomonads. We provide evidence that NCPPB 3753 and NCPPB 2877 form a unique species in the genus *Xanthomonas* herein called *Xanthomonas cannabis*. We further describe likely virulence traits encoded by their genomes. Most notably these strains lack a Hrp T3SS but possess the major *hrp* virulence regulators HrpX and HrpG. Based on our comparative genomic analysis in *X. cannabis*, we provide a putative model for acquisition of the T3SS, T3-secreted effectors and the *hrp* regulators in *Xanthomonas* spp.

## Results/methods/discussion

### Phenotypic evaluation

**Figure 1:**
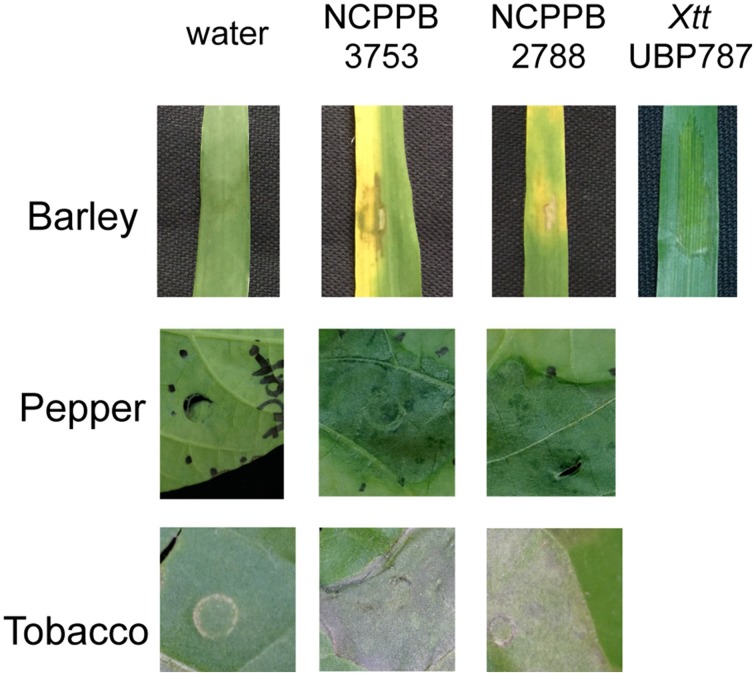
**Phenotypic analysis of** ***X. cannabis*** **on different plants**. Symptom development and HR were evaluated on barley, pepper, and tobacco. Leaves were inoculated by needleless infiltration with water-bacterial suspensions of *X. cannabis* NCPPB 3753, NCPPB 2877 (*O*D_600_ = 0.5) or water as a control. Images were taken 48 h post inoculation for barley and tobacco, or 4 days post inoculation for pepper. The barley pathogen, *X. translucens* pv. *translucens* strain UPB787, served as a positive control for symptoms on barley. Plants were grown in a growth chamber at 22°C, 50% humidity and 16 h of light.

Two representative strains of *X. cannabis* (also known as *Xanthomonas campestris* pv. *cannabis*), isolated from symptomatic hemp leaves (*C. sativa* L.), were chosen for genome sequencing. Type strain NCPPB 2877 was isolated by I. Sandru at the Lovrin station in the Timiş judeţ (Romania) in 1974 (Severin, 1978), and strain NCPPB 3753 was isolated by Y. Takiwawa in the Kanuma region of Tochigi Prefecture (Japan) in 1982 (isolate SUPP546; Netsu et al., 2014). *X. cannabis* strains were previously reported to cause disease on many dicot host plants. To determine if *X. cannabis* could infect a monocot host, we inoculated barley (*Hordeum vulgare* L. cv. Morex) leaves with the cannabis strains by infiltration. Overnight cultures grown using PSA (Tsuchiya et al., 1982) or NB medium (Sigma-Aldrich, USA) were pelleted and resuspended in water. Plant leaves were infiltrated by a needleless syringe with a water-bacterial suspension or water as a control. Leaves developed necrosis around the zone of infiltration followed by leaf yellowing (Figure 1). These symptoms closely resembled the leaf spot symptoms on *C. sativa* observed by previous characterizations (Severin, 1978; Netsu et al., 2014). Similar symptoms were observed with lower inoculum (*O*D_600_ = 0.05 and 0.1). Tomato is a compatible host for *X. cannabis* (Severin, 1978), and therefore we decided to test *X. cannabis* virulence of pepper, another solanaceous plant. Pepper leaves were infiltrated with strains NCPPB 2877 and NCPPB 3753 as with barley. Pepper plants displayed water-soaked lesions 48 h post inoculation (Figure 1). Both cannabis strains elicited an HR when inoculated on tobacco (Figure 1), but the nature of this HR remains to be determined.

### Genome sequencing and annotation

The genomes of strains NCPPB 2877 and NCPPB 3753 were sequenced using the Illumina Hi-Seq2500 platform (Fasteris SA, Switzerland). The shotgun sequencing yielded 2,921,175 100-bp paired-end reads (730 Mb) for strain NCPPB 2877 and 2,464,521 paired-end reads (616 Mb) for strain NCPPB 3753, with insert sizes ranging from of 250 bp to 1.5 kb. Draft genome sequences were assembled using the Edena algorithm v3.131028 (Hernandez et al., 2014), yielding 257 contigs ≥200 bp (*N*_50_ = 38,306 bp) with 69 × coverage for strain NCPPB 2877 and 260 contigs (*N*_50_ = 35,229 bp) with 73 × coverage for strain NCPPB 3753. For comparison, draft genome sequences were also assembled using the Velvet algorithm v1.1.04 (Zerbino and Birney, 2008), yielding 564 contigs ≥200 bp (*N*_50_ = 15,608 bp) for strain NCPPB 2877 and 469 contigs (*N*_50_ = 20,963 bp) for strain NCPPB 3753. Because of their better quality, Edena-derived contigs were annotated with GeneMarkS + release 2.9 (revision 452131) (Borodovsky and Lomsadze, 2014), as implemented in the NCBI Prokaryotic Genome Annotation Pipeline (http://www.ncbi.nlm.nih.gov/genome/annotation_prok/), which predicted a total of 4095 genes within 4,756,730 bp for strain NCPPB 2877 and 4160 genes within 4,837,471 bp for strain NCPPB 3753. These whole genome shotgun projects have been deposited at DDBJ/EMBL/GenBank under the accession no. JSZE00000000 (NCPPB 2877) and JSZF00000000 (NCPPB 3753). The versions described in this paper are the first versions, JSZE01000000 and JSZF01000000.

### Comparison of the two genome sequences

ANI provides a robust method to determine bacterial species definition based on whole genome sequence comparison and is considered the new standard for species definition (Konstantinidis and Tiedje, 2005; Figueras et al., 2014). To determine if NCPPB 2877 and NCPPB 3753 are the same species, the ANI was calculated for both genome sequences using JSpecies (Richter and Rosselló-Móra, 2009). BLAST-based comparison revealed 99.2% ANI for the 92.5% sequences that could be aligned, and MUMmer-based comparison revealed 99.1% ANI for the 96.9% sequences that could be aligned, thus confirming that both strains belong to the same species.

Using our web-based pipeline for prediction of satellites (http://www.biopred.net/VNTR/), we then evaluated whether or not both strains belong to a clonal complex. For satellite prediction, the following parameters were chosen (Zhao et al., 2012): algorithm, TRF; region length, 30–1000 bp; unit length, 5–12 bp; and at least 6 tandem repeats with a similarity of at least 80% among the repeats. In total, 45 microsatellites were predicted, 35 of which were found to be present in both genome sequences. For 34 of them, repeat numbers could be derived; while one locus was not informative because it was located at the end of two contigs and thus not completely assembled in NCPPB 3753. To provide further evidence that the calculated repeat numbers were meaningful, the corresponding loci were also analyzed in the Velvet-based genome assemblies. Strikingly, there was not a single discrepancy between the Edena- and Velvet-based data, except for the fact that some satellite loci were not completely assembled by Velvet while they were complete in the Edena assembly. For the complete loci, 28 loci (82%) were different between the two strains with respect to repeat numbers. For the six loci with identical repeat numbers DNA sequence analysis revealed that five of them were identical due to homoplasy, i.e., these loci evolved by convergent evolution to the same number of repeats. Thus, both strains differ by almost all (97%) of their completely assembled microsatellite loci, a finding that indicates that both strains do not belong to a clonal complex.

### Taxonomic position of the two cannabis pathogens

Comparison of 16S rDNA sequences is a method of choice to elucidate the taxonomic positions of bacterial strains, and was previously used to analyze and delineate 20 species of *Xanthomonas* (Hauben et al., 1997). It was found that the genus *Xanthomonas* exhibited a relatively high level of 16S rDNA sequence identity, with on average 14 single-nucleotide polymorphisms (SNPs) between two different *Xanthomonas* species (Hauben et al., 1997). The 16S rDNA sequences of both cannabis pathogens were found to be identical. When we compared the 16S rDNA sequence of the cannabis pathogens with those of the 20 *Xanthomonas* type strains, the cannabis pathogen grouped with Group 2 strains, which contains the majority of characterized *Xanthomonas* species. Interestingly, GenBank comparison revealed that another recently sequenced strain that was isolated from symptomatic bean plants in Rwanda, Nyagatare, contains the same 16S rDNA sequence (Aritua et al., 2015).

**Figure 2:**
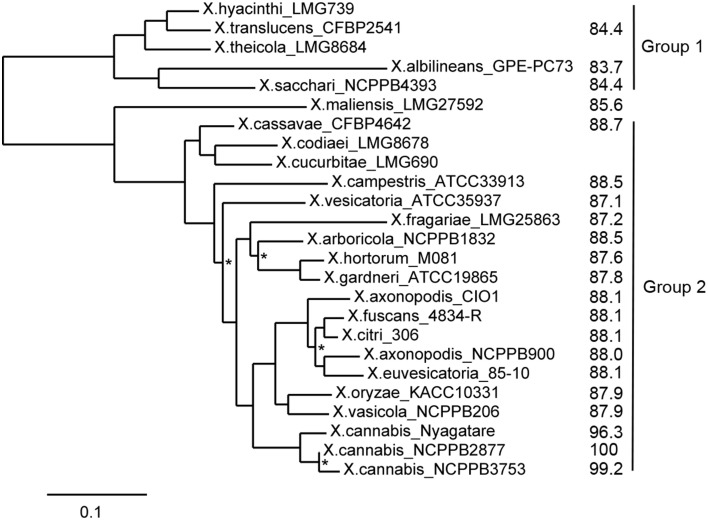
**ANI and MLSA-based phylogenetic tree of xanthomonads**. Phylogenetic analysis was performed on the Phylogeny.fr platform (Dereeper et al., 2008). Sequences were aligned with MUSCLE (v3.7) configured for highest accuracy (MUSCLE with default settings). After alignment, ambiguous regions (i.e., containing gaps and/or poorly aligned) were removed with Gblocks (v0.91b) using default parameters. The phylogenetic tree was reconstructed using the maximum likelihood method implemented in the PhyML program (v3.0). The HKY85 substitution model was selected assuming an estimated proportion of invariant sites and 4 gamma-distributed rate categories to account for rate heterogeneity across sites. The gamma shape parameter was estimated directly from the data. Reliability for internal branch was assessed using the aLRT test (SH-Like). Graphical representation and edition of the phylogenetic tree were performed with TreeDyn (v198.3). All nodes were supported by bootstrap values above 0.9, except for those marked with an asterisk. Calculated ANI values in comparison to NCPPB 2877 and the two major phylogenetic groups are indicated on the right side.

Previously *X. cannabis* strains were also called *X. campestris* pv. *cannabis* based on the similarity of the 16S rDNA sequence to *X. campestris*, but it has been suggested that the name should be changed to *X. cannabis* (Netsu et al., 2014). Since the resolution of the 16S rDNA sequence is very low within Group 2 strains (Hauben et al., 1997) and often only distinguishes a species by one or two SNPs, we performed whole-genome comparisons including one representative strain per species for which genome sequences were available (Figure 2). The pairwise ANI of the two cannabis strains against any of the representative strains was below 90%, regardless of which algorithm (BLAST or MUMmer) was used, indicating that these two strains belong to an unique and distinct *Xanthomonas* species (Figure 2). We suggest that *X. cannabis* is the appropriate name for this bacterial species based on our ANI analysis and as previously suggested by Netsu et al. (2014).

Guided by the observation that their 16S rDNA sequences were identical to that of the Nyagatare strain, we compared the genomes of *X. cannabis* NCPPB 3753 and NCPPB 2877 and *X*. sp. Nyagatare. JSpecies calculations revealed that the two cannabis strains were 96.3–96.4% identical to the Nyagatare strain, when calculated over the 88.6–91.0% of the genome sequence that could be aligned by the more robust MUMmer algorithm (Richter and Rosselló-Móra, 2009). These values are slightly above the ≈95–96% transition zone, above which strains can be considered to belong to the same taxonomically circumscribed prokaryotic species (Konstantinidis and Tiedje, 2005). Therefore the Nyagatare strain most likely belongs to the *X. cannabis* species. It would be interesting to perform functional studies to determine the similarities and differences between these closely related *X. cannabis* strains because the Nyagatare strain is a reported bean pathogen and *X. cannabis* strains elicit no response when inoculated on bean (Netsu et al., 2014).

Partial sequencing of the *gyrB* and other housekeeping genes for MLSA grouped all *Xanthomonas* species into four major MLSA subgroups (Parkinson et al., 2007; Young et al., 2008). We used MLSA of seven housekeeping genes (*atpD*, *dnaK*, *efp*, *glnA*, *gyrB*, *lepA*, and *rpoD*) and ANI calculations to understand the relationship of the cannabis and Nyagatare strains compared to other xanthomonads. The *X. cannabis* NCPPB 2877 and NCPPB 3753 and Nyagatare strains appear to form a distinct grouping according to MLSA (Figure 2). Based on the comparisons of ANI and of seven concatenated internal portions of the genes, we overall conclude that the cannabis and Nyagatare strains belong to a single species, *X. cannabis* because: (1) they are above the 95–96% ANI threshold for species definition and (2) they appear to belong to a new MLSA clade (Figure 2). This new clade corresponds to the novel species-level clade (slc) 1, as suggested by Parkinson and co-workers, which also contains the pathovars *esculenti* and *zinniae* (Parkinson et al., 2009). Following the pathovar designation, we suggest to name the two cannabis strains *X. cannabis* pv. *cannabis*, and the bean-pathogenic Nyagatare strain *X. cannabis* pv. *phaseoli*.

### Comparison of pathogenicity-related gene clusters

Several gene clusters are considered to be important for pathogenicity of xanthomonads and their possible contribution to host- and tissue-specificity has been studied previously (Lu et al., 2008). We therefore analyzed whether and to which extent these gene clusters are conserved in the cannabis strains.

The *rpf* (regulation of pathogenicity factors) gene cluster plays a role in the intercellular signal-response system that links synthesis and perception of the diffusible signal factor (DSF) cis-11-methyl-2-dodecenoic acid to the synthesis of extracellular enzymes, extracellular polysaccharide, and biofilm dispersal (Dow, 2008). The genetic organization of the cannabis *rpf* gene cluster resembles that of *X. euvesicatoria* strain 85–10, yet it contains an additional gene, *rpfI*, downstream of gene XCV1913, which is not present in *X. euvesicatoria* strain 85–10. Moreover, the proteins RpfF, responsible for DSF synthesis, and the two-component regulatory system RpfC/RpfG, which is involved in DSF perception and signal transduction (Dow, 2008), are highly conserved in the cannabis pathogens.

The *gum* gene clusters encode proteins that are involved in the exopolysaccharide (EPS) biosynthesis (Becker et al., 1998). The core *gum* gene cluster, consisting of *gumB* to *gumM*, all transcribed in the same direction, is entirely conserved in the cannabis pathogen. As in other xanthomonads, the first gene, *gumB*, is located downstream of a proline-specific tRNA gene. The *gumA* gene which is located upstream of the tRNA gene and downstream *pheS* and *pheT* is most likely not a *bona fide gum* gene. The accessory *gumN* gene, which for instance is disrupted in *X. euvesicatoria* strain 85–10 and *X. oryzae* pv. *oryzicola* strain BLS256, appears to be intact in the cannabis pathogen. *gumO* and *gumP*, annotated as a 3-oxoacyl-(acyl carrier protein) synthase and a metal-dependent hydrolase, respectively, are located directly downstream of *gumN*. The contribution of these two genes to EPS biosynthesis is questionable since it is not present in several xanthomonads infecting monocotyledons, such as *X. sacchari*, *X. translucens* and *X. oryzae*, and it is also not present in the recently sequenced *Xanthomonas maliensis* strain 97M, which was isolated from rice leaves in Mali. On the other side, this unique conservation in bacteria colonizing eudicots could suggest a role in host specificity at the level of a division.

Lipopolysaccharide (LPS) is another bacterial polysaccharide, which is firmly attached to the outer membrane, and an aberrant structure of the LPS O-chain has been linked to virulence defects (Mhedbi-Hajri et al., 2011). The gene cluster responsible for LPS biosynthesis is always present between the highly conserved *etfA* and *metB* genes of *Xanthomonas*. A remarkably high degree of variation both in number and in identity of LPS genes has been found in different xanthomonads, even within a single species or pathovar (Patil et al., 2007). In the two cannabis pathogens, 14 genes are predicted to participate in the LPS biosynthesis. The gene content and genetic organization is largely identical to a few other *Xanthomonas* strains belonging to different species: *Xanthomonas gardneri* strain ATCC 19865, *Xanthomonas vesicatoria* strain ATCC 35937, and strains of *X. campestris* (B100, CN14, CN15, CN16, JX, Xca5, and 756C); except that the *X. campestris* strains have a short, additionally predicted gene, *wxcH*, just downstream of the *etfA* gene, which is not present (or frame-shifted) in the other species. Interestingly, the relatively closely related Nyagatare strain shares only the four *etfA*-proximal genes, *wxcK* to *wxcN*, and the two *metB*-proximal genes, *wzm* and *wzt*, with the cannabis strains, while between them three large genes without orthologs in other xanthomonads are predicted. As observed before on a smaller set of *Xanthomonas* genome sequences (Patil et al., 2007; Lu et al., 2008), the cannabis pathogens underscore the fact that the LPS gene cluster varies highly between and within species, suggesting multiple horizontal gene transfers and re-assortments. Yet, it is unclear what evolutionary pressure drives this variation—is it escape from recognition by plant immune receptors, and/or is it escape from recognition by bacteriophages?

**Figure 3:**
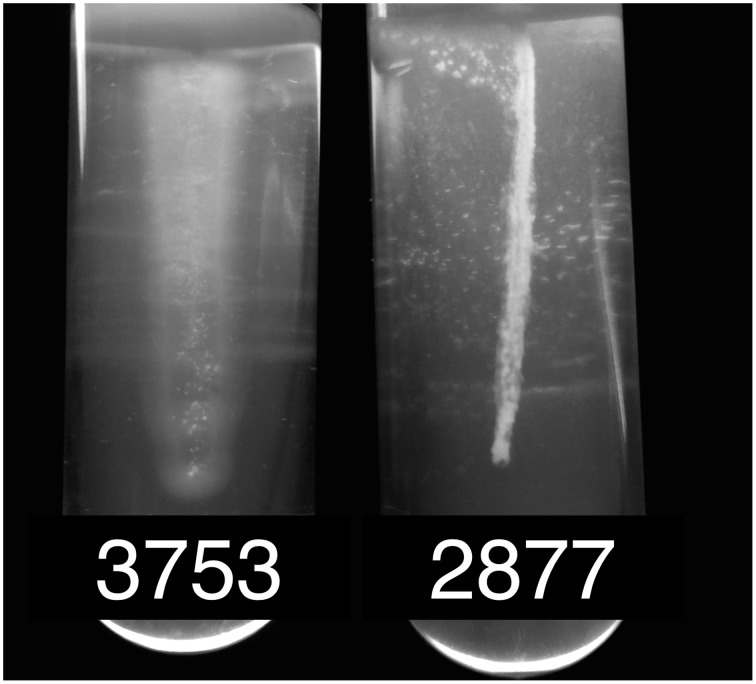
**Motility of** ***X. cannabis*** **strains**. Bacterial motility was determined by stab inoculation in motility medium of either strain NCPPB 3753 or NCPPB 2877. Motility was evaluated qualitatively by turbid growth (motile) compared to localized, fixed growth (non-motile) around the zone of inoculation. Bacteria were grown in semi-solid NYGA agar medium (0.3%) as previously described (Sun et al., 2006).

Motility is an important feature of bacteria that is governed by flagella-based swimming and/or pilus-based twitching or gliding (Rossez et al., 2015). The flagellum is a complex machinery, the biosynthesis of which depends on dozens of genes, collectively called *fle*, *flg*, *flh*, and *fli* genes, which are organized in large gene clusters. The cannabis strain NCPPB 2877 contains the full gene complement of flagellar biosynthesis. In contrast, strain NCPPB 3753 appears to have lost two large regions in its two genes clusters. One deletion encompasses the genes *flgG* to *flgJ* and the 5′ portion of *flgK*. Downstream of *flgF*, remnants of an IS element were present in the genome assembly. The second, even larger deletion goes from the 3′ portion of *fliK* until the end of *flhA*, totaling to more than 11 genes. Absence of flagella under standard culture conditions was confirmed for strain NCPPB 3753 by a swimming assay in semisolid medium (Figure 3). Briefly overnight cultures grown in liquid NB medium were fixed to an *O*D_600_ = 1.0 and stabbed into semi-solid NYGA medium (0.3% agar). Motility was defined as observed turbid growth outside the stab zone of inoculation in the semi-solid medium. Tubes inoculated with NCPPB 3753 were turbid after 4 days of growth (Figure 3), which demonstrates that this strain is motile. Growth of NCPPB 2877 was confined to the stab zone of inoculation (Figure 3), which supports our hypothesis that NCPPB 2877 is non-motile because it lacks a flagellum.

Loss of flagella in xanthomonads is not without precedent (Darrasse et al., 2013). When Darrasse and co-workers tested 300 *Xanthomonas* strains representing different species and pathovars, five percent of the tested strains turned out to carry a deletion in the flagellar cluster and were non-motile. Co-isolation of flagellate and non-flagellate variants from an outbreak suggested that flagellar motility is not essential for fitness within the plant and that mixed populations could be a strategy to avoid detection by the plant defense system (Darrasse et al., 2013). Indeed, many plants have evolved receptor-like kinases (e.g., FLS2) that detect peptide epitopes of the FliC subunit of the flagellum (e.g., flg22 and flgII-28) (Sun et al., 2006), and it has been reported that otherwise isogenic flagellin-deficient *Xanthomonas* bacteria have a colonization advantage over flagellated bacteria in citrus leaves (Shi et al., 2015). Interestingly, when we analyzed the FliC flagellin sequence of the two cannabis strains, we detected a flg22 variant that deviates from the consensus sequence by as much as nine amino acid residues and is identical to the flg22 sequences from other xanthomonads, such as *X. oryzae* and *X. vasicola* (Supplemental Figure S1). In contrast, the related Nyagatare strain has a flg22 sequence that only deviates from the consensus by four residues and is similar to the elicitation-active flagellins that are recognized by FLS2 in Arabidopsis (Sun et al., 2006). These findings suggest that the cannabis strains use at least two out of several strategies to escape from recognition by the plant immune surveillance system, either by loss of the flagellum or by allelic variation of flagellin epitope(s) (Rossez et al., 2015).

The cannabis pathogen contains two different Type II protein secretion systems, the Xcs and the Xps system, as found in several other xanthomonads (Lu et al., 2008). The *xcs* gene cluster, consisting of 12 genes, *xcsC* to *xcsN*, is located downstream of a TonB-dependent receptor gene and upstream of a GntR-family regulator gene, which is also the case for *X. euvesicatoria* strain 85–10 and strains of *X. campestris*. The related Nyagatare strain contains another four genes between *xcsN* and the GntR-family regulator gene, a feature that is shared with the *X. citri* pv. *citri* strain 306 and the *Xanthomonas arboricola* strain 3004. Interestingly, the *xcs* gene cluster is also present in the *X. maliensis* strain 97M, but absent in the Group 1 strains *X. albilineans* GPE PC-73, *X. sacchari* NCPPB 4393, and *X. translucens* pv. *cerealis* CFBP 2541. The second Type II secretion system, encoded by the *xps* gene cluster, consists of 11 genes, *xpsE* to *xpsN*, followed by *xpsD*, and is found between the adhesion gene *xadA* and the *pnuC* gene. This position in the bacterial chromosome is largely conserved among *Xanthomonas* strains belonging to different Groups, such as the *X. sacchari* strain NCPPB 4393, the *X. translucens* pv. *cerealis* strain CFBP 2541, the *X. maliensis* strain 97M, and strains of Group 2.

Many Gram-negative plant-pathogenic bacteria have evolved another protein secretion system, the T3SS, which plays a pivotal role in the pathogen-host interaction (Büttner, 2012). The Hrp system serves as a molecular syringe that injects T3 effector proteins into the cytosol of the host's cells at the benefit of the bacterium. Yet, many injected effectors can also betray the pathogen to the host plant, triggering a strong defense response, which typically is accompanied by an effector-triggered HR (White et al., 2009). Notably the early-branching Group 1 sugarcane pathogen *X. albilineans* does not have an Hrp T3SS, a finding that could be attributed to the reduced genome size of this pathogen (Pieretti et al., 2009). Since then, new genome sequences became available, including other Group 1 strains belonging to the species *X. sacchari* (Studholme et al., 2011). Some of these strains do not appear to have undergone extensive genome reduction, yet, they do not encode a T3SS. When we analyzed the gene content of the cannabis strains we realized that they do not have any of the *hrc*, *hrp*, or *hpa* genes encoding the T3SS. We were also not able to detect any traces of a T3-effector gene when using the set of described genes as queries (http://www.xanthomonas.org/t3e.html). In contrast, the Nyagatare strain has a full gene complement for the Hrp T3SS and also possesses a couple of effector proteins (Aritua et al., 2015). This is an interesting finding for several reasons. First, how do the cannabis pathogens suppress plant defense, a function that became more and more linked to the activity of T3-effectors (e.g., Canonne et al., 2011; Schulze et al., 2012; Sinha et al., 2013; Li et al., 2015; Stork et al., 2015)? Second, which molecular entities are triggering the HR in *Nicotiana tabacum* if not type III effectors? Interestingly, a type II-secreted pectate lyase, XagP, from *X. axonopodis* pv. *glycines* was previously found to be associated with HR induction on tobacco and pepper, but not on cucumber, sesame and tomato, thus giving a prime example that cell wall-degrading enzymes could trigger an HR (Kaewnum et al., 2006). We therefore speculate that similar or other secreted enzymes that disturb the integrity of the plant cell wall could be responsible for HR triggered by the cannabis pathogen independent of a T3SS. Last but not least, these Group 2 genome sequences may serve as a valuable resource to predict new T3 effectors. As a relative of other xanthomonads it will provide a valuable negative training or filtering set for prediction algorithms.

### Analysis of the HrpX regulon

Previous work has shown that the Hrp T3SS and many of its secreted effectors are controlled by a regulatory cascade, consisting of HpaR2/HpaS, HrpG, and HrpX (Büttner and Bonas, 2010; Li et al., 2014). However, HrpG and HrpX regulate pathogenesis beyond the T3SS alone (Guo et al., 2011). We therefore wondered if these components are present in *Xanthomonas* strains that do not possess the Hrp T3SS, and if so, what genes would be controlled by these components. BLAST searches revealed that all four regulatory genes are conserved in the cannabis pathogens (Table 1). *hpaR2* and *hpaS* are also conserved in the *X. maliensis* strain and in strains from Group 1, such as *X. albilineans*, *X. sacchari* and *X. translucens*. Their location is always between the *glmS* and *glmU* gene, with some extra genes for an efflux pump between *glmS* and *hpaR2*. In contrast, *hrpG* and *hrpX* were not found in strains of *X. albilineans* or *X. sacchari*, but they are present in *X. translucens*. Interestingly, however, *hrpG* and *hrpX* from *X. translucens* cluster together with the rest of the T3SS *hrp* gene cluster while all other xanthomonads have *hrpG* and *hrpX* at a distinct genomic location between *radA* and *hsp70* (Wichmann et al., 2013). We could not determine their genomic organization for *X. maliensis* due to the highly fragmented genome assembly.

**Table 1:** **Presence of type III secretion systems, type III effectors,** ***hrp/hpa*** **regulatory genes, and homologs of predicted HrpX regulon members of** ***X. cannabis*** **pv**. ***cannabis*** **in representative strains of** ***Xanthomonas***.

**Group**	**Species**	**Strain**	**GenBank accession no**.	**Hrp T3SS**	**T3Es**	**HrpG HrpX**	**HpaS HpaR2**	**PehA**	**PehD**	**Amino-peptidase**	**LPL**
1	*X. albilineans*	GPE PC73	FP565176	no	no	no	YES	YES	YES	no	YES
1	*X. sacchari*	NCPPB 4393	AGDB01000001	no	no	no	YES	YES	no	YES	YES
1	*X. translucens* pv. *cerealis*	CFBP 2541	JWHD01000001	YES	YES	YES	no^a^	YES	YES	Ψ	YES
	*X. maliensis*	97M	AQPR01000001	no	no	YES	YES	no	PIP	PIP[5]	PIP[1]
2	*X. campestris* pv. *campestris*	ATCC 33913	AE008922	YES	YES	YES	YES	PIP	PIP[1]	PIP[1]	PIP
2	*X. vesicatoria*	ATCC 35937	AEQV01000004	YES	YES	YES	YES	PIP	PIP	PIP[1]	PIP
2	*X. vasicola* pv. *vasculorum*	NCPPB 206	AKBM01000001	YES	YES	YES	YES	PIP	no	PIP[1]	PIP
2	*X. oryzae* pv. *oryzae*	KACC110331	AE013598	YES	YES	YES	no^b^	Ψ	YES	no	PIP
2	*X. fuscans* subsp. *fuscans*	4834-R	FO681494	YES	YES	YES	YES	PIP	PIP	PIP[1]	PIP
2	*X. citri* pv. *citri*	306	AE008923	YES	YES	YES	YES	PIP	PIP	PIP[1]	PIP
2	*X. axonopodis* pv. *manihotis*	CIO1	AKCZ01000001	YES	YES	YES	YES	PIP	no	PIP[1]	PIP-Ψ
2	*X. axonopodis* pv. *vasculorum*	NCPPB 900	JPHD01000001	YES	YES	YES	no^c^	PIP	PIP	PIP[1]	PIP
2	*X. euvesicatoria*	85-10	AM039952	YES	YES	YES	YES	PIP	PIP	PIP[1]	PIP
2	*X. cassavae*	CFBP 4642	ATMC01000001	YES	YES	YES	YES	PIP	PIP	PIP[2]	PIP[3]
2	*X. arboricola* pv. *celebensis*	NCPPB 1832	JPHC01000001	YES	YES	YES	YES	PIP	no	PIP[1]	PIP[1]
2	*X. hortorum* pv. *carotae*	M081	AEEU01000001	YES	YES	YES	YES	PIP	PIP	PIP[1]	PIP
2	*X. gardneri*	ATCC 19865	AEQX01000001	YES	YES	YES	YES	PIP	PIP	PIP[1]	PIP
2	*X. fragariae*	LMG 25863	AJRZ01000001	YES	YES	YES	YES	PIP	PIP	Ψ	Ψ
2	*X. cannabis* pv. *phaseoli*	Nyagatare	JRQI01000001	YES	YES	YES	YES	PIP	PIP	PIP	PIP
2	*X. cannabis* pv. *cannabis*	NCPPB 2877	JSZE01000001	no	no	YES	YES	PIP	PIP	PIP	PIP
2	*X. cannabis* pv. *cannabis*	NCPPB 3753	JSZF01000001	no	no	YES	YES	PIP	PIP	PIP	PIP

*“YES” indicates presence of a homolog, “no” indicates absence of the protein(s)*.

*“Ψ” indicates pseudogenes, i.e., the gene is split into two or more fragments*.

*“PIP” indices the presence of the gene along with a canonical PIP box and a properly spaced −10 promoter motif. Numbers in square brackets indicate the number of single-nucleotide variants with respect to the canonical PIP box and −10 promoter motif*.

a *hpaR2 is eroded while an N-terminally truncated form of hpaS is present in X. translucens pv. cerealis strain CFBP 2541. Remnants are found in two other X. translucens strains (ART-Xtg27 and DSM 18974). Interestingly, the hpaR2/hpaS locus is intact in X. translucens strain DAR61454. This is an example of ongoing gene erosion in one species of Xanthomonas*.

b *The hpaR2/hpaS locus got destroyed by an IS element in X. oryzae pv. oryzae strain KACC 10331. Yet, hpaR2/hpaS is present in X. oryzae pv. oryzicola strain BLS256. Remnants are found in the African X. oryzae pv. oryzae strain NAI8 and in X. oryzae strains from the United States. This finding illustrates ongoing gene erosion in another species of Xanthomonas*.

c *hpaR2 is absent while an N-terminally truncated form of hpaS is present in X. axonopodis pv. vasculorum strain NCPPB 900*.

Within the Hpa-Hrp regulatory cascade, HrpX is the most downstream component that directly induces the synthesis of pathogenicity factors, such as the Hrp T3SS, T3 effectors and cell wall-degrading enzymes, by binding to a conserved *cis* element, called PIP (plant-induced promoter) box, within the promoter regions of the corresponding genes (Koebnik et al., 2006). For gene activation, both the PIP box as well as a properly spaced −10 promoter motif are required (Furutani et al., 2006). We therefore analyzed the genome sequences of the cannabis pathogens for the presence of the promoter motif TTCGB-N_15_-TTCGB-N_30−32_-TYNNNT (B represents C, G, or T; Y represents C or T) (Koebnik et al., 2006). Using this conservative query, four genes were strongly predicted to belong to the HrpX regulon, two polygalacturonases (PehA, synonymous with PghAxc, and PehD, synonymous with PghBxc and PglA; Hsiao et al., 2008; Wang et al., 2008), one putative aminopeptidase and one putative lysophospholipase (LPL). Indeed, the two polygalacturonases have been previously shown to be regulated by HrpX in *X. campestris* (Wang et al., 2008). For *X. citri*, microarray transcriptome studies have shown previously that three genes with a canonical PIP box and −10 promoter motif (PehA, PehD, LPL; Table 1) are positively regulated by HrpX (Guo et al., 2011). Finally, our own RNAseq data show that the LPL is under control of HrpX in an African *X. oryzae* strain (unpublished data). We highly suspected that the four predicted genes with a canonical PIP box and properly spaced −10 promoter motif were indeed under control of HrpX in the cannabis strains.

Genome mining revealed that these four genes are present in most xanthomonads from Groups 1 and 2 (Table 1). Yet, in some lineages, one or the other gene apparently got lost. For instance, *X. sacchari*, *X. maliensis*, *X. arboricola*, *X. axonopodis* pv. *manihotis*, *X. oryzae*, and *X. vasicola* lack one or the other polygalacturonase, and the aminopeptidase is absent from *X. albilineans* and *X. oryzae*. In a few cases, these genes appear to have suffered from pseudogenization (Table 1) but this needs confirmation by targeted DNA sequencing due to the risk of sequence errors in some of the draft genome sequences.

**Figure 4:**
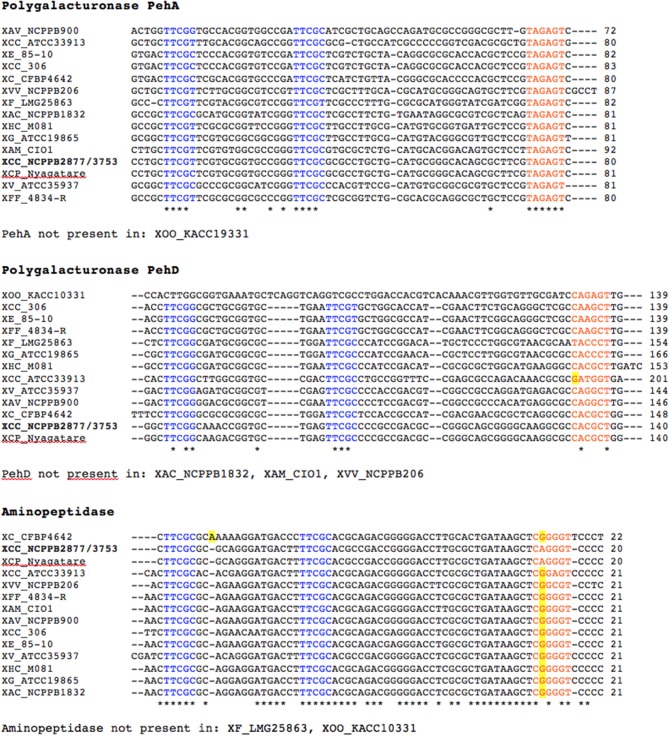
**Promoter sequences of predicted HrpX regulon members and their homologs in strains of** ***Xanthomonas***. Promoter regions encompassing 350 bp upstream of the translational start codon of a representative set of *Xanthomonas* strains were aligned by MUSCLE. PIP half boxes are shown in blue and the −10 promoter motif is shown in orange. Distance to the translational start codon is indicated on the right side of each sequence block. Deviations from the PIP consensus sequence are highlighted in yellow. The following *Xanthomonas* strains were analyzed: XAC (*X. arboricola* pv. *celebensis*) NCPPB 1832, XAM (*X. axonopodis* pv. *manihotis*) CIO1, XAV (*X. axonopodis* pv. *vasculorum*) NCPPB 900, XCC (*X. campestris* pv. *campestris*) ATCC 33913, XCC (*X. cannabis* pv. *cannabis*) NCPPB 2877 and NCPPB 3753, XCP (*X. cannabis* pv. *phaseoli*) Nyagatare, XC (*X. cassavae*) CFBP 4642, XCC (*X. citri* pv. *citri*) 306, XE (*X. euvesicatoria*) 85-10, XF (*X. fragariae*) LMG 25863, XFF (*X. fuscans* subsp. *fuscans*) 4834-R, XG (*X. gardneri*) ATCC 19865, XHC (*X. hortorum* pv. *carotae*) M081, XOO (*X. oryzae* pv. *oryzae*) KACC 19331, XVV (*X. vasicola* pv. *vasculorum*) NCPPB 206, and XV (*X. vesicatoria*) ATCC 35937. ^*^Denotes conserved nucleotide.

Since these four genes appear to be under control of HrpX in the cannabis pathogens, we looked for evidence that the same regulation occurs in the other xanthomonads. We therefore compared the upstream regions of these four genes in a representative set of *Xanthomonas* strains. Strikingly, we found PIP boxes and properly spaced −10 promoter motifs for most of the genes in most of the *Xanthomonas* strains (Figure 4, Supplemental Figure S2), except for the Group 1 strains *X. albilineans*, *X. sacchari*, and *X. translucens*. Multiple sequence alignments of the promoter regions of the three genes show that the PIP boxes are conserved in sequence, context and position (PIP boxes ~140–150 bp before start codon of *pehA*, ~90 bp before start codon of the aminopeptidase gene, and ~210–250 bp before start codon of *pehD*) indicate that the PIP boxes of the polygalacturonase and aminopeptidase genes evolved early after separation of Group 2 from Group 1. In contrast, the PIP boxes of the lysophospholipase gene do not align with each other and reveal four subgroupings, which are compatible with a MLSA-based phylogeny of Group 2 strains (Supplemental Figure S2). This finding could suggest that the PIP box evolved several times independently at early times after separation of the four MLSA subgroups, or that the surrounding sequences evolved too extensively to allow the PIP boxes to be aligned using standard parameters.

**Figure 5:**
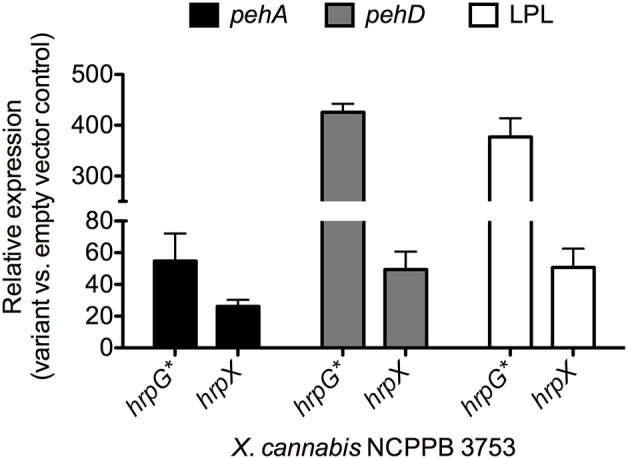
**Gene expression analysis of genes with HrpX-inducible PIP boxes**. Bacteria were grown overnight in liquid NB (Sigma Aldrich, USA) supplemented with gentamycin (20 μg l^−1^) and transferred to fresh 10 mL NB media with gentamycin for a final *O*D_600_ = 0.5. Bacteria were incubated for 3 h, shaking at 28°C. Transcriptional profiles and RNA was preserved with 5% phenol in ethanol as previously described (Jahn et al., 2008; Jacobs et al., 2012). Bacterial RNA was extracted with Trizol (Invitrogen, USA), cleaned up with Zymogen RNA concentrator (Zymo Research, USA) and treated with Turbo DNase (Invitrogen, USA) following manufacturer's protocols. RNA (1 μg per sample) was reverse transcribed with Superscript III (Invitrogen, USA) following the manufacturer's recommendation. qPCR with SYBR MESA BLUE Master Mix (Eurogentec, Belgium) was performed following the manufacturer's protocol on a Roche LightCycler 480 Real-Time PCR instrument (Roche Diagnostics Corporation, USA) with reaction parameters of 10-min polymerase activation at 95°C, then 40 cycles, with an individual cycle consisting of 15 s at 95°C and 1 min at 60°C. Relative gene expression analysis of *pehA*, *pehD* and the gene encoding the putative lysophospholipase (LPL) were calculated by the delta-delta-Ct method (Livak and Schmittgen, 2001) for NCPPB 3753 variants ectopically expressing hrpG^*^ or *hrpX* compared to NCPPB 3753 pBBR1MCS-5. Expression of *atpD* was used as a normalization internal control gene. Primers are listed in Supplemental Table S1.

To test if HrpX and HrpG could activate expression of the putative targets, we quantified gene expression of *pehA*, one of the four genes with a PIP box, in various mutant backgrounds of *X. cannabis* NCPPB 3753. HrpG^*^ from *X. euvesicatoria* 85–10 is a HrpG variant that mimics the active form of HrpG (Wengelnik et al., 1999). Mutant strains with hrpG^*^ induce expression of *hrpX* and further genes targeted by HrpX, which includes primarily promoters with PIP boxes (Wengelnik et al., 1999). We therefore transformed *X. cannabis* cells with either pBBR1MCS-5 (empty vector), pBBR1MCS-5::*hrpX* or pBBR1MCS-5::hrpG^*^, as previously described (Koebnik et al., 2006). hrpG^*^ from *X. euvesicatoria* was cloned into pBBR1MCS-5, as described for *hrpX* (Wengelnik et al., 1999; Koebnik et al., 2006). Positive transformants were confirmed by PCR (data not shown). Quantitative PCR (qPCR) was used to determine the relative expression of putative HrpX-target genes in *X. cannabis* NCPPB 3753 pBBR1MCS-5::hrpG^*^ and NCPPB 3653 pBBR1MCS-5::*hrpX* compared to NCPPB 3753 pBBR1MCS-5 (empty vector) as a control. *atpD* was used as a normalization control. *pehA*, *pehD*, and the gene encoding the putative LPL were dramatically induced in NCPPB 3753 ectopically expressing hrpG^*^ or *hrpX*, compared to the empty vector control (Figure 5). NCPPB 3753 pBBR1MCS-5::hrpG^*^ specifically upregulated (fold-change [SE]) *pehA* (54.8 ± 17.3), *pehD* (425.8 ± 16.6) and *LPL* (377.1 ± 36.5) compared to the empty vector control. A similar trend was observed with expression of the three genes (26.2 ± 4.1, 49.4 ± 11.3, 50.7 ± 11.9, respectively) in NCPPB 3753 pBBR1MCS-5::*hrpX* compared to the empty vector control. We did not detect differential expression of the putative aminopeptidase in our *hrp* regulatory variant backgrounds under the conditions mentioned above (data not shown). We only tested one set of primers for this gene under one condition, and further experiments need to be performed to determine if this negative result suggests that this gene is not a target of HrpX. We overall conclude that the promoter of *pehA*, *pehD*, and *LPL* are *bona fide* targets of HrpX, validating our bioinformatic analysis. This supports the hypothesis that HrpG and HrpX regulate pathogenesis beyond T3SS alone (Tang et al., 2006). Further investigation of the role of HrpX and HrpX targets remains to be performed, but *X. cannabis* is a potentially interesting model to understand the role of HrpX beyond the T3SS.

One of the polygalacturonase genes, *pehA*, is not only under control of HrpX but was found to be regulated by Clp and RpfF in *X. campestris* (Hsiao et al., 2008). Since Clp and RpfF are conserved over all clades it is tempting to speculate that this layer of regulation has evolved before separation of the clades. However, comparison of the *pehA* promoter regions in a representative set of *Xanthomonas* strains revealed that the predicted Clp-binding site is not conserved (Supplemental Figure S3). Notably, multiple sequence alignment instead revealed a conserved sequence motif the position of which is shifted by 8 bp with respect to the predicted Clp-binding site (Supplemental Figure S3). In fact, this conserved box is as similar to the canonical Clp box as is the previously predicted Clp-binding site (Dong and Ebright, 1992), and its position is compatible with the mapping of the Clp-binding site by *lacZ* reporter fusions (Hsiao et al., 2008). Probably, the Clp-binding site was slightly mispredicted at that time due to the absence of sufficient sequence data from diverse *Xanthomonas* strains, which help to uncover regulatory DNA elements.

## Conclusions

**Figure 6:**
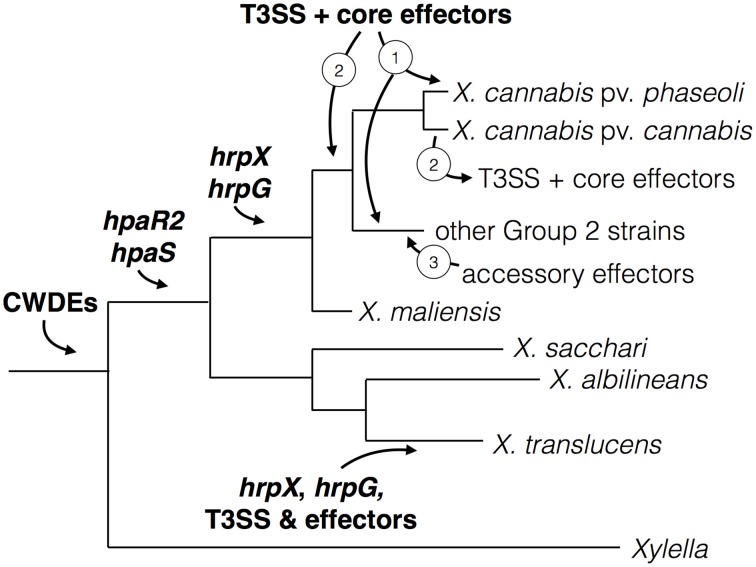
**Model for virulence gene and regulator acquisition in** ***X. cannabis***. The acquisition of various virulence traits was likely sequential in *Xanthomonas* spp., which represented by a schematic phylogenetic tree. Cell-wall modification by cell-wall degrading enzymes (CWDEs) is an ancient trait found in both *Xylella* and *Xanthomonas*, which suggests their acquisition predates the separation of these two pathogenic genera. Genes encoding the regulators HpaR2 and HpaS were subsequently acquired before the separation of the *Xanthomonas* Group 2 and early branching Group 1. Then the regulatory genes *hrpX* and *hrpG* were gained by Group 2 strains and further promoters (e.g., PIP boxes) evolved to adapt to these virulence regulators. This is further supported by the absence of PIP boxes in front of non-*hrp* genes in Group 1 strains (*X. albilineans* and *X. sacchari*). Because the organization of *hrpX* and *hrpG* and the T3SS is more similar to *R. solanacearum*, different than Group 2 xanthomonads and lacking in most Group 1 species (*X. sacchari* and *X. albilineans*), we suspect an independent acquisition of this system in *X. translucens*. *hrpG* and *hrpX* are present in a similar location in all sequenced Group 2 species. We hypothesize that the T3SS and core effectors were either (1) acquired independently by individual pathovars or (2) acquired an earlier point and lost in some pathovars. After the acquisiton of the T3SS, we posit that accessory effectors were acquired (3) by horizontal gene transfer to alter host range and/or promote susceptibility by suppressing plant immunity.

Stimulated by two new genome sequences from cannabis-pathogenic xanthomonads, we explored the world of pathogenicity determinants in the genus *Xanthomonas*. A plethora of research data as well as our own analyses let us speculate about a stepwise evolution of pathogenicity in *Xanthomonas*. We developed a model (Figure 6) of evolution and acquisition of *Xanthomonas* pathogenicity factors based on a model proposed by Lu et al. (2008). Given the observation that some *Xanthomonas* strains across distinct taxa do not contain *hrpG* and *hrpX*, we speculate a stepwise evolution of pathogenicity (Figure 6), which involves (i) acquisition of key regulatory genes and cell wall-degrading enzymes, followed by (ii) acquisition of the Hrp type III secretion system, which is ultimately accompanied by (iii) successive acquisition of type III effectors. In parallel, as soon as *hrpG* and *hrpX* were acquired, a subset of genes, which can contribute to pathogenicity, evolved PIP boxes in their promoter regions thus ensuring their efficient expression during plant colonization.

Basic pathogenicity factors, such as the Xps type II secretion system and its secreted cell wall-degrading enzymes, as well as the *rpf* gene cluster, were probably already present in the ancestor of *Xanthomonas* and *Xylella*. Perhaps, these components were already expressed in response to environmental conditions, as we can still observe for the genes that are controlled by RpfF and/or Clp. When the xanthomonads had separated into distinct genetic clades (Group 1 vs. Group 2), *hrpG* and *hrpX* were acquired, and perhaps evolved to be cross-regulated by the HpaR2/HpaS two-component regulatory system. It is conceivable that the *X. translucens* lineage acquired *hrpX* and *hrpG* together with the Hrp type III secretion system since the two regulatory genes are physically linked to the *hrp* gene cluster (Wichmann et al., 2013), reminiscent of the situation in *Ralstonia solanacearum*. Notably, the other early-branching xanthomonads, such as *X. albilineans* and *X. sacchari*, did not acquire *hrpX* nor *hrpG*. In contrast, all other xanthomonads have the oppositely transcribed *hrpG* and *hrpX* genes in the same synteny group between *radA* and *hsp70*, suggesting a unique acquisition event.

Interestingly, there are xanthomonads that possess *hrpG* and *hrpX* but not the Hrp Type III secretion system nor any type III effectors, such as the cannabis pathogen, the new *X. maliensis* clade (Triplett et al., 2015), and a recently sequenced strain of *X. arboricola* (Ignatov et al., 2015). This could be taken as evidence that the Hrp system was either (1) acquired later or (2) got lost in some lineages. We favor the hypothesis that the T3SS and core effectors were gained in the case of the Nyagatare strain and not lost from *X. cannabis* NCPPB 2788 and NCPPB 3753. There seems to be no indication of any remnant features of the T3SS or effectors in the two *X. cannabis* genomes. Moreover, the Nyagatare strain possesses the T3SS and only core effectors, which appear to be located on a pathogenicity island flanked by insertion/transposable elements. More systematic genome sequencing of underexamined genetic lineages will shed light on this evolutionary puzzle.

### Conflict of interest statement

The authors declare that the research was conducted in the absence of any commercial or financial relationships that could be construed as a potential conflict of interest.

## Supplementary material

Supplemental Figure S1 **Flg22 epitope variants found in various strains of *Xanthomonas***. On top, the prototype elicitor-active flg22 peptide from *Pseudomonas aeruginosa* is shown (Felix et al., 1999). Below, homologous sequences from different species and pathovars of *Xanthomonas* are aligned. Residues that deviate from the prototype sequence are in red. The peptide that corresponds to eliciting flagellin variants of *X. campestris* pv. *campestris* is highlighted in green, while the peptide that corresponds to non-eliciting flagellin variants highlighted in yellow (Sun et al., 2006).

Supplemental Figure S2 **Comparison of promoter sequences of the *Xanthomonas* lysophospholipase gene**. Promoter regions encompassing 350 bp upstream of the translational start codon of a representative set of *Xanthomonas* strains were aligned by MUSCLE. PIP half boxes are shown in blue and the −10 promoter motif is shown in orange. Distance to the translational start codon is indicated on the right side of the lower sequence block. Deviations from the PIP consensus sequence are highlighted in yellow. For the set of analyzed strains, compare with Figure 4.

Supplemental Figure S3 **Comparison of promoter sequences of the *Xanthomonas pehA* gene**. Promoter regions encompassing 350 bp upstream of the translational start codon of a representative set of *Xanthomonas* strains were aligned by MUSCLE. Consensus CAP/CLP binding boxes, according to Dong and Ebright (1992), are indicated above the multiple sequence alignment, first aligned with the Clp-binding site as determined by Hsiao et al. (2008), for *X. campestris* pv. *campestris* (highlighted in yellow, six conserved residues are shown in green), and then aligned with the Clp-binding site as proposed by us (conserved residues are shown in red). The PIP half boxes are shown in blue and the −10 promoter motif is shown in orange. Distance to the translational start codon is indicated on the right side of the lower sequence block. For better comparison with Figure 4, PIP half boxes are shown in blue and the −10 promoter motif is shown in orange. Distance to the translational start codon is indicated on the right side of the lower sequence block. Deviations from the PIP consensus sequence are highlighted in yellow. For the set of analyzed strains, compare with Figure 4.
