# Supplementary figures and images for: Comparative genomics of a cannabis pathogen reveals insight into the evolution of pathogenicity in Xanthomonas
Source: Front Plant Sci. 2015 Jun 16;6:431. doi: 10.3389/fpls.2015.00431 (PMC4468381; doi:10.3389/fpls.2015.00431)

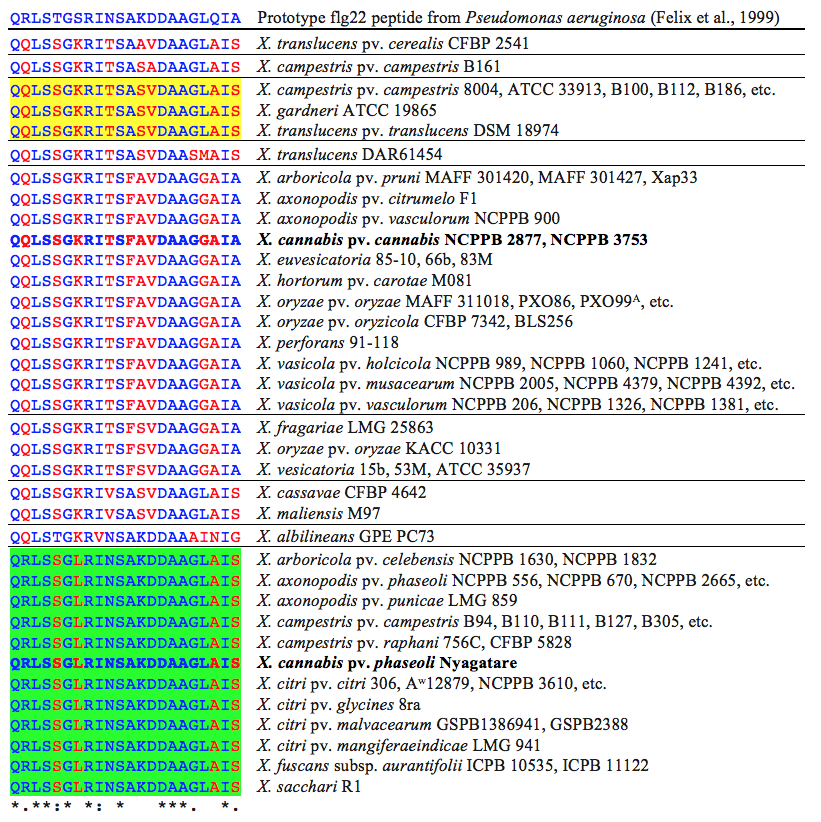

Supplement: Supplemental Figure S1 — Flg22 epitope variants found in various strains of Xanthomonas. On top, the prototype elicitor-active flg22 peptide from Pseudomonas aeruginosa is shown (Felix et al., 1999). Below, homologous sequences from different species and pathovars of Xanthomonas are aligned. Residues that deviate from the prototype sequence are in red. The peptide that corresponds to eliciting flagellin variants of X. campestris pv. campestris is highlighted in green, while the peptide that corresponds to non-eliciting flagellin variants highlighted in yellow (Sun et al., 2006). [file Image1.TIFF]

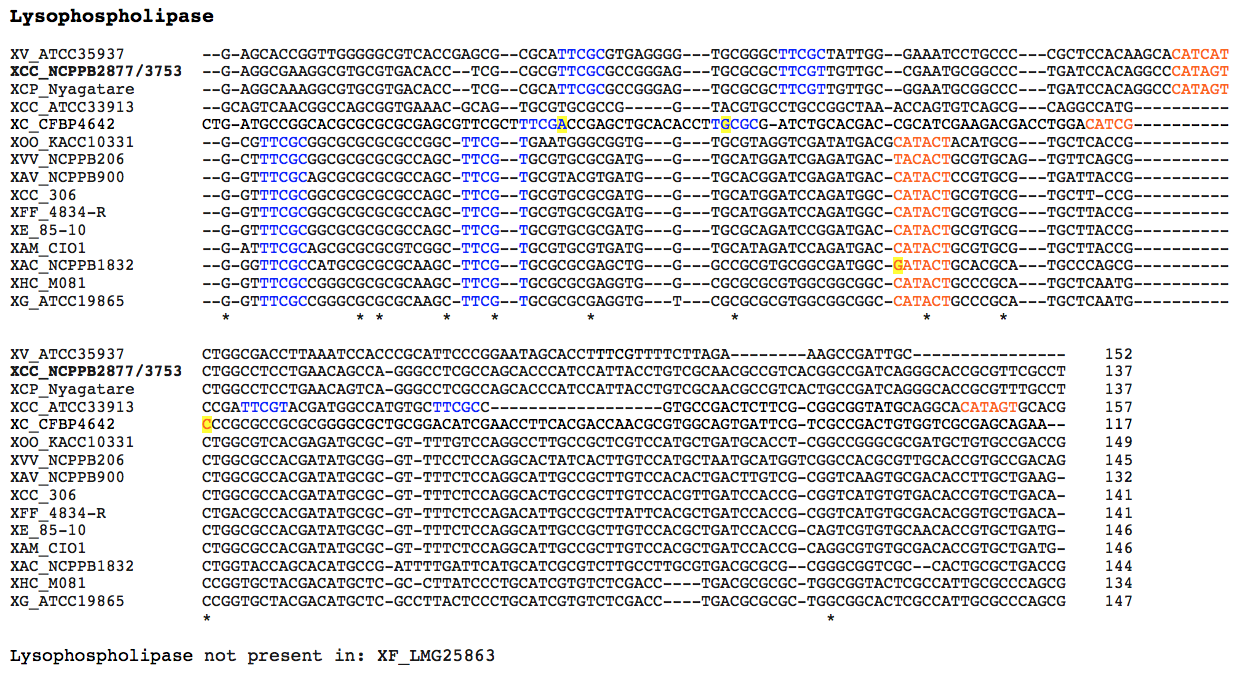

Supplement: Supplemental Figure S2 — Comparison of promoter sequences of the Xanthomonas lysophospholipase gene. Promoter regions encompassing 350 bp upstream of the translational start codon of a representative set of Xanthomonas strains were aligned by MUSCLE. PIP half boxes are shown in blue and the −10 promoter motif is shown in orange. Distance to the translational start codon is indicated on the right side of the lower sequence block. Deviations from the PIP consensus sequence are highlighted in yellow. For the set of analyzed strains, compare with Figure 4. [file Image2.TIFF]

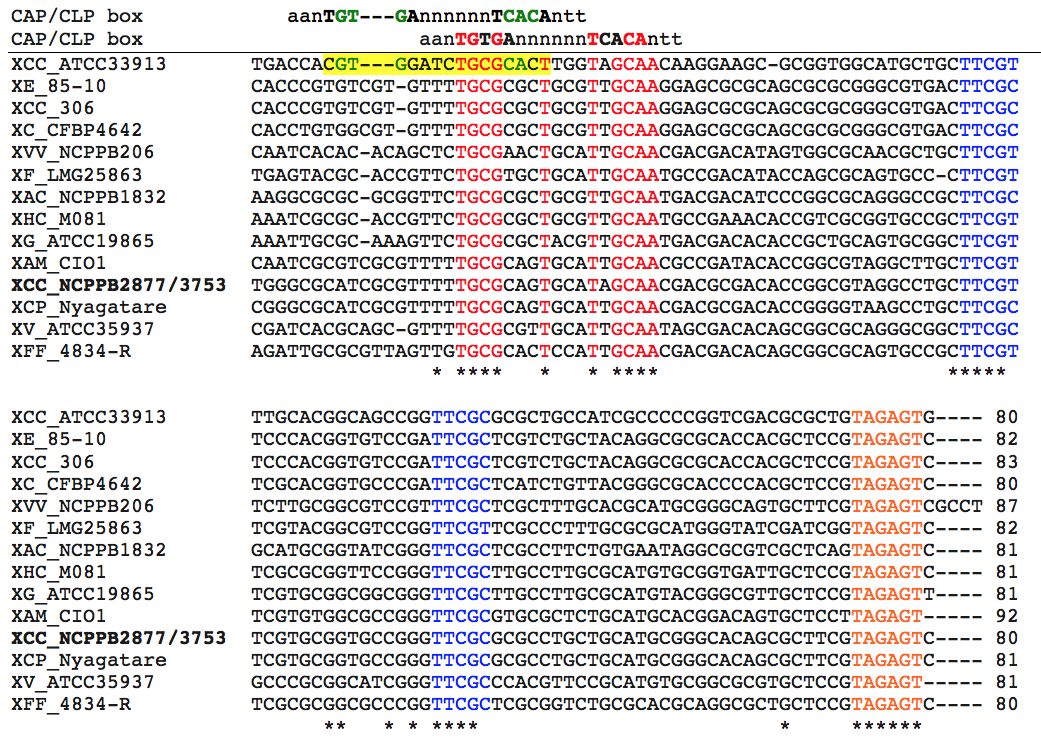

Supplement: Supplemental Figure S3 — Comparison of promoter sequences of the Xanthomonas pehA gene. Promoter regions encompassing 350 bp upstream of the translational start codon of a representative set of Xanthomonas strains were aligned by MUSCLE. Consensus CAP/CLP binding boxes, according to Dong and Ebright (1992), are indicated above the multiple sequence alignment, first aligned with the Clp-binding site as determined by Hsiao et al. (2008), for X. campestris pv. campestris (highlighted in yellow, six conserved residues are shown in green), and then aligned with the Clp-binding site as proposed by us (conserved residues are shown in red). The PIP half boxes are shown in blue and the −10 promoter motif is shown in orange. Distance to the translational start codon is indicated on the right side of the lower sequence block. For better comparison with Figure 4, PIP half boxes are shown in blue and the −10 promoter motif is shown in orange. Distance to the translational start codon is indicated on the right side of the lower sequence block. Deviations from the PIP consensus sequence are highlighted in yellow. For the set of analyzed strains, compare with Figure 4. [file Image3.TIFF]
