# Supplementary material for: Comparative genomics of a cannabis pathogen reveals insight into the evolution of pathogenicity in Xanthomonas
Source: Front Plant Sci. 2015 Jun 16;6:431. doi: 10.3389/fpls.2015.00431 (PMC4468381; doi:10.3389/fpls.2015.00431)
Supplement: Supplementary file 4 [file Table1.PDF]

Supplemental Table S1. Primers used for qPCR.

Primer	Sequence	Gene target
2JMJ_0053	CAACCATGTGTTCGTCGCTG	*pehA* (Forward)
2JMJ_0054	CTGAGTGCCGCCTGGATG	*pehA* (Reverse)
2JMJ_0055	AATCGCTCACCACCGAAGTG	Lysophospholipase (Forward)
2JMJ_0056	CGGTCCACACCTTGTCGAAG	Lysophospholipase (Reverse)
2JMJ_0059	CGACCTGTTTTACCCCGGAA	*pehD* (Forward)
2JMJ_0060	GCCGATGTACGAATAGGCCA	*pehD* (Reverse)
2JMJ_0065	GAACGCGCCATTTCGGTG	*atpD* (Forward)
2JMJ_0066	GTCACCGGCTTCGTCGAT	*atpD* (Reverse)
